# A perspective of immunotherapy for acute myeloid leukemia: Current advances and challenges

**DOI:** 10.3389/fphar.2023.1151032

**Published:** 2023-04-19

**Authors:** Ying Chen, Jishi Wang, Fengqi Zhang, Ping Liu

**Affiliations:** ^1^ Department of Hematology, Affiliated Hospital of Guizhou Medical University, Guiyang, China; ^2^ Guizhou Province Institute of Hematology, Guizhou Province Laboratory of Hematopoietic Stem Cell Transplantation Centre, Guiyang, China

**Keywords:** acute myeloid leukemia, immunotherapy, checkpoint inhibitor, car-t, DC vaccines

## Abstract

During the last decade, the underlying pathogenic mechanisms of acute myeloid leukemia (AML) have been the subject of extensive study which has considerably increased our understanding of the disease. However, both resistance to chemotherapy and disease relapse remain the principal obstacles to successful treatment. Because of acute and chronic undesirable effects frequently associated with conventional cytotoxic chemotherapy, consolidation chemotherapy is not feasible, especially for elderly patients, which has attracted a growing body of research to attempt to tackle this problem. Immunotherapies for acute myeloid leukemia, including immune checkpoint inhibitors, monoclonal antibodies, dendritic cell (DC) vaccines, together with T-cell therapy based on engineered antigen receptor have been developed recently. Our review presents the recent progress in immunotherapy for the treatment of AML and discusses effective therapies that have the most potential and major challenges.

## Introduction

Acute myeloid leukemia (AML) is a form of leukemia that mostly affects the adult population with most cases occurring in this age group and is diagnosed less frequently in children. First-line chemotherapy has been the mainstay of treatment during the last four decades (Dohner et al., 2010). Despite initial response to first-line treatment and disappearance of symptoms for most patients, only a small fraction achieves prolonged survival due to chemo-resistant relapses. Superior survival has been observed in the patient group undergoing allogeneic hematopoietic stem cell transplantation (allo-HSCT), but these patients only constitute a small fraction of AML cases (Ferrara and Schiffer, 2013; Kadia et al., 2015; Coombs et al., 2016; Short et al., 2018), and the relapse does not seem to decrease with graft-versus-host disease as well as patients with active disease (Vago and Gojo, 2020). Patients who have never achieved complete remission (CR) or relapse within 6 months after achieving a CR have poorer prognosis (De Kouchkovsky et al., 2016).

Immune surveillance is pivotal in suppressing tumor growth and maturation. The immune system can identify the antigens specific to the tumor cells. Nevertheless, the tumor microenvironment fosters immunosuppressive activities and antigen loss that would eventually lead to immune evasion. On the one hand, T-cell surface checkpoint regulators such as cytotoxic T lymphocyte-associated antigen-4 (CTLA-4) and programmed cell death protein 1 (PD-1) have been demonstrated to contribute, at least in part, to these immunosuppressive activities (Gurusamy et al., 2017; Mohme et al., 2017). Enhancement of inhibitory receptors confers robust immune effector response (Deng et al., 2018). Based on this immunosuppression mechanism, CTLA-4 and PD-1 checkpoint blockade therapies have shown outstanding clinical efficacy in killing tumor cells in several solid organ malignancies (Tang et al., 2017). The continued success of immunotherapy in solid tumors has prompted its use in hematologic malignancies, and PD-1 blockade has shown promising preliminary results in classical Hodgkin lymphoma (cHL) (Hodi et al., 2010; Topalian et al., 2012; Robert et al., 2014; Younes et al., 2016). However, patients with other hematological malignancies, such as AML, have shown fewer encouraging results, underlining the need for future studies in these tumor types. On the other hand, to prevent antigen loss, a novel class of immune therapy strategies has been developed, including specific antibodies, dendritic cell (DC) vaccines, chimeric antigen receptor-engineered T cells (CAR-T), T-cell receptor-engineered T cells (TCR-T). Several antibody-based therapies have become the paradigm for treating acute lymphoblastic leukemia (ALL), and several others are being evaluated in clinical studies. Addition of Rituximab, a CD20-directed cytolytic antibody, to first line chemotherapy has demonstrated enhanced efficacy (Levato and Molica, 2018). Autologous CAR-T cell therapy targeting CD19 has shown striking responses, leading to FDA’s approval for the management of precursor B-cell ALL and diffuse large B-cell lymphoma (DLBCL) (Grupp et al., 2013).

Immune checkpoint inhibitors are capable of activating tumor-specific immune cells that are suppressed in the tumor microenvironment (TME). For hematological malignancies such as AML with a maturation and progression pattern different from solid tumors, the mechanisms underlying the activity or inactivity of the immune system may strikingly differ from previous descriptions for solid tumors. Despite the presence of mutual immune evasion pathways, hematological tumors have their unique tolerance mechanisms (Zhang et al., 2009; Zhou et al., 2009; Zhou et al., 2011; Mussai et al., 2013). Moreover, checkpoint blockade has demonstrated greater efficacy in tumors with higher mutation burdens (Ansell et al., 2015; Chalmers et al., 2017; Yarchoan et al., 2017). Characterized by a low mutation burden, AML has only 13 genetic mutations on average in newly diagnosed disease (Cancer Genome Atlas Research et al., 2013), which mean that the immune system has a smaller chance to recognize mutated coding sequences giving rise to neoantigens (Gettinger et al., 2017; Haanen, 2017; Yarchoan et al., 2017). Thus, a better insight into the mechanisms through which immune evasion occurs in AML is fundamental to help evaluate different immunologic strategies for this disease based on study data. In CAR-T therapy, genetically modified immune cells possess new tumor-targeting specificity and efficacy. While the restricted CD19 and CD20 expression profile of ALL patients facilitates successful targeting of these B cell-associated antigens, the heterogeneous tumor antigen expression in a wide array of AML makes it more difficult to select an suitable target antigen (Taussig et al., 2005; Levine et al., 2015). Leukemogenesis has been understood better in recent years (Schwartzman and Tanay, 2015; Greaves, 2016; Ferrando and Lopez-Otin, 2017), and specifically, newly discovered molecular markers have yielded new insight into the pathogenesis of AML. An increasing number of potential markers have been investigated for every immunotherapeutic strategy (Perna et al., 2017; Yang and Wang, 2018). Nevertheless, further clinical research is necessary to ascertain the clinical efficacy.

In the present review, recent advances with regard to immunotherapies for AML will be discussed, various challenges in the field will be looked at, and emerging strategies that may optimize treatment efficacy will be presented.

## Checkpoint inhibitor therapy

**FIGURE 1:**
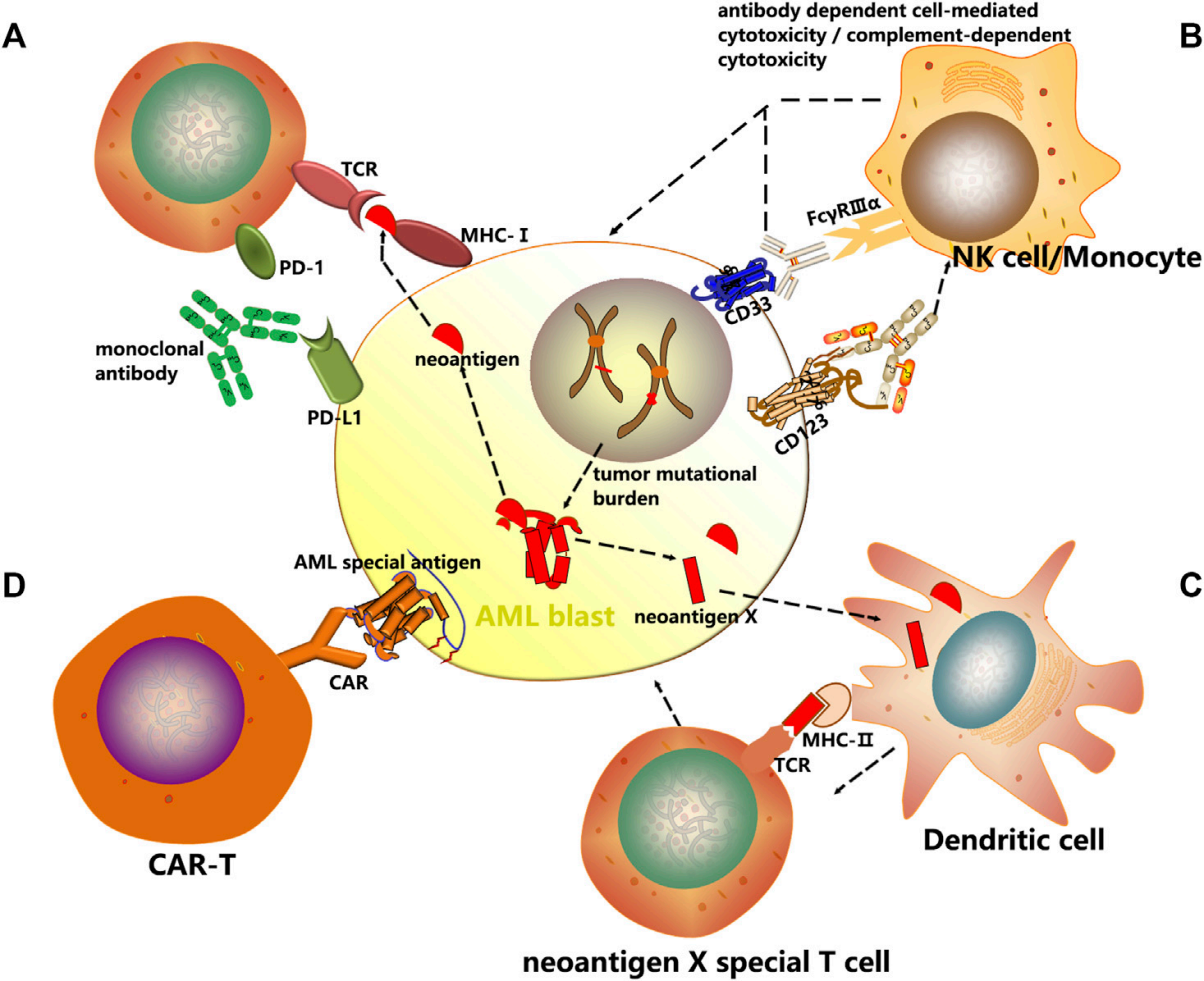
Mechanisms of cancer immunotherapy. In this review, several immunotherapeutic strategies have been discussed with an emphasis on AML treatment. **(A)** Checkpoint inhibitors are a new class of immunotherapy that use monoclonal antibodies to modulate inhibitory receptors on T cells to enhance T cell-mediated immune responses. **(B)** The antibody dependent cell mediated cytotoxicity (ADCC) and complement dependent cytotoxicity (CDC) induced by AML surface antigen-directed antibodies, as well as the effect of an internalized antibody-toxin conjugate result in AML cell death. **(C)** Dendritic cells are unique antigen-presenting cells (APC) and comprise the main class of APC. Autologous CD14^+^ monocytes derived from peripheral blood of cancer patients are differentiated with GM-CSF and IL-4/IL-13 into immature DCs *in vitro*. Thereafter immature DCs are supplied with tumor antigens, that is, whole cell lysates, tumor-associated antigens (TAAs) or neoantigens. These antigen-loaded DCs are further matured and then enhance vaccine-induced immune responses. **(D)** Chimeric antigen receptors (CARs) are genetically modified receptor proteins with both extracellular antigen binding domain and intracellular signaling domain. With their unique structure, particularly effective cytotoxic activity, as well as antigen binding in an MHC-independent fashion is possible.

Targeting immune checkpoints such as CTLA4, PD1 and PD-L1 has produced impressive results across a diverse variety of malignancies including AML through immunoinhibitory signal blockage and CD8^+^ T-cell activation to initiate a strong antitumor response (Figure 1A).

## CTLA-4

CTLA-4 is an immune inhibitor mainly found on the surface of T cells. Through engaging the co-stimulatory protein CD28, ligation of CTLA-4 and CD80/CD86 occurs. CTLA-4 out competes CD28 and exhibits greater affinity in binding CD80 and CD86, which results in inhibiting T cells (Friedman et al., 2016; Byun et al., 2017; Nishino et al., 2017). The higher expression of CTLA-4 on T cells was detected in peripheral blood from patients with newly diagnosed AML (Chen et al., 2020a). As the first immunosuppressive molecule identified, CTLA-4 has provided an optional treatment approach in addition to conventional chemotherapy. Ipilimumab and tremelimumab as CTLA-4 inhibitors were used to treat patients with different cancers in clinical trials and led to prolonged overall survival (Author Anonymous, 2010; Hodi et al., 2010; Kyriakidis et al., 2021), but with limited off-label use in AML. In several clinical studies, favorable anti-leukemic effect, anti-tumor reactivity for relapsed patients and durable responses were reported (Fevery et al., 2007; Davids et al., 2016). Thus, it is suggested that hematological malignancies be treated with the use of CTLA-4 inhibitor. Ipilimumab produced specific potency in treating relapsed AML patients after HSCT (NCT01822509) (Liao et al., 2019; Vago and Gojo, 2020; Penter et al., 2021) and holds promise in affecting local control for the treatment of AML-related extramedullary (Bakst et al., 2020). However, patients with AML have less response to ipilimumab. Moreover, the rational combinations has potential to improve the efficacy of drugs and ipilimumab plus nivolumab have been approved for the management of metastatic melanoma and several other malignancies (Rotte, 2019). Notwithstanding, the two drugs, when used in combination, showed no improving effect on AML (Greiner et al., 2020) and was more likely to cause infectious complications (Spallone et al., 2022). Non-etheless, clinical investigation is ongoing to determine the potent effects of CTLA-4 inhibitor for AML patients (Masarova et al., 2017).

## PD-1/PD-L1

As a potent immune checkpoint protein, PD-1 receptor acts like a “brake” on the immune response (Rizvi et al., 2015; Wolchok, 2015; Im et al., 2016; Gordon et al., 2017; Kamphorst et al., 2017; Kelly, 2017; Eroglu et al., 2018). Blocking this checkpoint by PD-1 inhibitors has resulted in impressive clinical response rates in melanoma (Roboz et al., 2014) and non-small cell lung cancer (Giroux Leprieur et al., 2017), as well as in Hodgkin (Le et al., 2015; Armand et al., 2018) and non-Hodgkin lymphomas (Lesokhin et al., 2016).

AML is characterized by upregulated PD-1 levels expressed on T cells, which is closely related to the treatment and prognosis of the disease (Huang et al., 2019; Tan et al., 2020; Tang et al., 2020). Patients newly diagnosed with AML or those undergoing relapse reported elevated PD-1 levels in peripheral blood (PB) and bone marrow (BM) T cells following front-line treatment, and by contrast, patients in durable remission reported reduced expression levels (Xu et al., 2021a; Brauneck et al., 2021), and TCRβ sequencing revealed clonal expansion in PD-1 positive CD8^+^ T cells (Feng et al., 2020). The effector function in the BM milieu was reactivated through PD-1, CTLA-4, or TIM3 immune checkpoint pathways (Lamble et al., 2020). PD-L1/PD-L2 are found on the surface of leukemic cells, as demonstrated in several studies (Chen et al., 2008; Berthon et al., 2010). Driver mutations could regulate the expression of inhibitory proteins, for example, PD-L1/PD-L2 overexpression shown in AML cells with NRAS and ASXL1 mutations (You et al., 2022). Similarly, upregulation of PD-L1 was noted in patient cohorts with myelodysplastic syndromes (MDS) harboring TP53 mutations and secondary AML (sAML) (Sallman et al., 2020). Moreover, MDS or AML patients treated with hypomethylating agents (HMA) have also shown elevated PD-1 and PD-L1 expression in peripheral blood mononuclear cells (Yang et al., 2014). The mechanism of immune escape induced by PD-L1 may directly drive Treg cell expansion (Dong et al., 2020). PD-L1 silencing led to significant IFN-γ secretion and proliferation of MiHA-specific CD8+T cells, which was equally effective as PD-1 antibody blockade (van Ens et al., 2020).

Now six PD-1/L1 inhibitors were approved for treatment of lung cancer, melanoma, breast cancer or lymphoma including nivolumab (Opdivo^®^), pembrolizumab (Keytruda^®^), atezolizumab (Tecentrip^®^), avelumab (Bavencio^®^), durvalumab (Imfinzi^®^), and cemiplimab (Libtayo^®^). Immune checkpoint inhibitors (ICIs) confer advantages for solid tumors that are yet to be attained for AML (Chen et al., 2020b). However, the successful therapeutic application of ICIs therapy in solid tumors has inspired its use in hematological malignancies, and the favorable clinical responses achieved in Hodgkin lymphoma are promising. Currently, PD-1 therapy is only applied for patients with relapsed/refractory AML in clinical trials. An increasing number of investigational studies are in progress to assess the efficacy of the anti-PD-1 inhibitor nivolumab. The relapsed AML patients after allo-HSCT showed good response when treated with nivolumab but paid attention to the clinical signs of graft versus-host disease (GVHD) (Albring et al., 2017; Yao et al., 2021). Another study found that HSCT with prior use of nivolumab and/or ipilimumab appears feasible in patients with AML and cyclophosphamide-based GVHD prevention is associated with improved prognosis (Oran et al., 2020).

As we mentioned earlier, combined strategies produced clinical benefits for the treatment of AML. Daver et al. reported promising preliminary results in a study combining azacitidine and nivolumab for adult cohort with relapsed and refractory AML (*n* = 51) (Naval Daver et al., 2016; Daver et al., 2018). Another phase II study also demonstrated the safety and efficacy of azacitidine plus nivolumab in patients with AML in first salvage status, with HMA naïvety, or with increased CD3^+^ BM infiltration as assessed by flow cytometry or IHC (Daver et al., 2019). The combination of the two drugs had an acceptable tolerability and produced a favorable response rate in relapsed AML patients, which is associated with the potential mechanism of adaptive T-cell plasticity and genomic alterations (Abbas et al., 2021). In addition, another clinical trial (NCT02397720) is recruiting patients to determine the therapeutic effect of azacitidine and nivolumab with ipilimumab or without ipilimumab in AML patients who were not responsive or relapsed and newly diagnosed elderly patients (>65 years). In a phase II trial (NCT02464657), the use of three drugs (nivolumab, idarubicin and cytarabine) in combination has been proved efficacious in subjects who were newly diagnosed with AML or at high risk of MDS (Ravandi et al., 2019). Moreover, several clinical studies reported the combined effect of azacitidine and other inhibitors on refractory AML, such as pembrolizumab (Zeidner et al., 2021) and avelumab (Saxena et al., 2021) with encouraging clinical activity. The therapy of pembrolizumab combined with decitabine was clinically feasible in patients who were not responding well or relapsed (NCT02996474) (Goswami et al., 2022). Given the small cohort likely to benefit from ICI therapy, further investigation is necessary to explore the mechanism of action. However, not all combinations can improve rates of durable responses for AML patients. The use of avelumab in combination with decitabine in a phase I clinical trial achieved no clinical benefit in AML patients (Zheng et al., 2021), as well as the combination of ipilimumab and nivolumab (Greiner et al., 2020). Nevertheless, several additional trials are ongoing across the globe to assess the clinical efficacy of ICIs combined with induction chemotherapy or hypomethylating agents for immunotherapy of AML (Table 1).

**TABLE 1:** Current clinical trials using checkpoint inhibitors for immunotherapy of AML.

Trial identifier	Study name	Conditions	Target	Drug name	Clinical phases	Enrollment	Start date	Completion date	Status
NCT02532231	Nivolumab in Acute Myeloid Leukemia (AML) in Remission at High Risk for Relapse	AML	PD-1	Nivolumab	Ⅱ	30	2015	2022	Active, not recruiting
NCT02275533	Nivolumab in Eliminating Minimal Residual Disease and Preventing Relapse in Patients With Acute Myeloid Leukemia in Remission After Chemotherapy	AML in Remission	PD-1	Nivolumab	Ⅱ	82	2015	2023	Active, not recruiting
NCT03092674	Azacitidine With or Without Nivolumab or Midostaurin or Decitabine and Cytarabine Alone in Treating Older Patients With Newly Diagnosed Acute Myeloid Leukemia or High-Risk Myelodysplastic Syndrome	AML	PD-1	Nivolumab	Ⅱ/Ⅲ	1670	2017	2023	Recruiting
		MDS							
NCT02846376	Single Agent and Combined Inhibition After Allogeneic Stem Cell Transplant	AML	PD-1	Nivolumab Ipilimumab	Ⅰ	8	2019	2023	Active, not recruiting
		MDS							
NCT03825367	Nivolumab in Combination With 5-azacytidine in Childhood Relapsed/Refractory AML	AML, Childhood	PD-1	Nivolumab	I/II	26	2019	2024	Recruiting
NCT02397720	Nivolumab and Azacitidine With or Without Ipilimumab in Treating Patients With Refractory Relapsed or Newly Diagnosed Acute Myeloid Leukemia	AML	PD-1	Nivolumab	Ⅱ	182	2015	2022	Recruiting
		Recurrent AML		Ipilimumab					
NCT03600155	Nivolumab and Ipilimumab After Donor Stem Cell Transplant in Treating Participants With High Risk Refractory or Relapsed Acute Myeloid Leukemia	Allo-HSCT Refractory AML	PD-1	Nivolumab	Ⅰ	55	2018	2022	Recruiting
				Ipilimumab					
NCT02845297	Study of Azacitidine in Combination With Pembrolizumab in Relapsed Refractory Acute Myeloid Leukemia (AML) Patients and in Newly Diagnosed Older (>65 Years) AML Patients	AML	PD-1	Pembrolizumab	Ⅱ	67	2016	2022	Active, not recruiting
NCT02771197	Lympho depletion and Anti-PD-1 Blockade to Reduce Relapse in AML Patient Not Eligible for Transplant	AML	PD-1	Pembrolizumab	Ⅱ	20	2016	2023	Active, not recruiting
NCT02768792	High Dose Cytarabine Followed by Pembrolizumab in Relapsed Refractory AML	Relapse AML	PD-1	Pembrolizumab	Ⅱ	38	2016	2024	Active, not recruiting
NCT03286114	Augmentation of the Graft vs Leukemia Effect Via Checkpoint Blockade With Pembrolizumab	MDS	PD-1	Pembrolizumab	Ⅰ	20	2017	2022	Recruiting
		AML							
		ALL							
NCT02981914	Pilot Study of Pembrolizumab Treatment for Disease Relapse After Allogeneic Stem Cell Transplantation	AMLetc.	PD-1	Pembrolizumab	Ⅰ	26	2017	2029	Recruiting
NCT04284787	BLAST MRD AML-2: BLockade of PD-1 Added to Standard Therapy to Target Measurable Residual Disease in Acute Myeloid Leukemia 2- A Randomized Phase 2 Study of Anti-PD-1 Pembrolizumab in Combination With Azacitidine and Venetoclax as Frontline Therapy in Unfit Patients With Acute Myeloid Leukemia	AMLetc.	PD-1	Pembrolizumab	II	76	2020	2023	Recruiting
NCT04372706	RTX-240 Monotherapy and in Combination With Pembrolizumab	Solid Tumor	PD-1	Pembrolizumab	I/II	166	2020	2023	Not yet recruiting
		AML Adult							
NCT04353479	Combined PD1 Inhibitor and Decitabine in Elderly Patients With Relapse and Refractory Acute Myeloid Leukemia	AML	PD-1	Camrelizumab (SHR-1210)	II	29	2020	2022	Not yet recruiting
NCT04722952	PD-1 Inhibitor Combined With Azacytidine and Homoharringtonine, Cytarabine, G-CSF for Refractory or Relapsed AML	Refractory Leukemia	PD-1	Visilizumab	III	30	2021	2024	Recruiting
		Relapse adult AML							
		AML							
NCT02935361	Guadecitabine and Atezolizumab in Treating Patients With Advanced Myelodysplastic Syndrome or Chronic Myelomonocytic Leukemia That Is Refractory or Relapsed	MDS	PD-L1	Atezolizumab	Ⅰ/Ⅱ	33	2016	2024	Active, not recruiting
		Recurrent AML With MDS							
NCT03390296	Pfizer Immunotherapy Combinations for Acute Myeloid Leukemia (AML) Multi-Arm Study 1	Recurrent AML	PD-L1	Avelumab	Ⅱ	138	2017	2024	Active, not recruiting
		Refractory AML							
NCT02890329	Ipilimumab and Decitabine in Treating Patients With Relapsed or Refractory Myelodysplastic Syndrome or Acute Myeloid Leukemia	Recurrent AML	CTLA-4	Ipilimumab	Ⅰ	48	2017	2023	Active, not recruiting

In addition to CTLA-4 and PD-1/PD-L1, numbers of clinical trials of TIM-3 inhibitors including sabatolimab and TQB2618 for AML are recruiting patients (NCT05426798, NCT03940352, NCT04623216, NCT05367401, NCT03066648). Similarly, TIM-3 inhibitors combined with demethylation drugs (azacitidine or decitabine) is used as the treatment strategy for AML, and one of the clinical trial adopts the combination of spartalizumab (PD-1 inhibitor) and sabatolimab (NCT03066648), which indicates that TIM3 inhibitors will occupy the market in the field of AML in the future.

## Monoclonal antibodies therapy

Recent studies have identified numerous candidate AML-associated antigens that can be targeted, including CD33 and CD123. By selectively targeting AML specific antigens, monoclonal antibodies have emerged as effective therapeutic agents that can reduce morbidity and mortality. During treatment of AML, monoclonal antibodies can exert their therapeutic effects through selective drug delivery, cell-mediated and complement-mediated mechanisms, and innate immune enhancement. Besides, antibody-drug conjugation combining chemotherapeutic agents or radioactive particles with monoclonal antibodies is also being evaluated to specifically target cells expressing CD33 or CD123. Despite that the immune system is not activated, they can induce cell injuries which may initiate the innate immune system and activate immune defense (Figure 1B).

## Anti-CD33

CD33 is a Siglec receptor protein primarily expressed on leukemic blasts. CD33 expression alone cannot be used as an independent prognostic marker, but low expression of CD33 is often observed in groups with complex karyotypes and translocations, and *FLT3-ITD* or *NPM1* mutations are more prevalent in groups with higher CD33 expression level (Feldman et al., 2005; Pollard et al., 2012; Krupka et al., 2014; Khan et al., 2017). In addition, the endocytosed CD33 makes it a promising target in antibody drug conjugates (ADCs) therapy (Krupka et al., 2014; Laszlo et al., 2016).

The first approved ADC by the FDA is gemtuzumab ozogamycin (GO/Mylotarg) targeting CD33 in 2000 to treat AML (Bross et al., 2001) and was subsequently removed from market due to increased risk of hepatic veno-occlusive disease (VOD) and high mortality (Petersdorf et al., 2013). However, cytotoxic therapy utilizing CD33 has attracted enormous attention because of the remarkable responses in varying patient cohorts receiving GO. New data from a fractionated-dosing schedule for treating newly diagnosed and relapsed/refractory AML led to re-approval of GO (Jen et al., 2018). This was supported by the results that demonstrating decreased number of early deaths, bleeding, low platelet count and VOD while sustaining treatment efficacy (Baron and Wang, 2018). In the phase III ALFA-0701 trial, fractionated doses of GO in combination with daunorubicin and cytarabine (DA) chemotherapy improved event-free survival (EFS) compared to chemotherapy alone (17.3 vs 9.5 months). Moreover, a complete remission (26%) was achieved with GO in relapsed/refractory AML (Lambert et al., 2019). The use of azacitidine and GO in combination in a phase I/II trial for relapsed AML showed good tolerability and achieved a CR of 24% in 12/50 patients (Medeiros et al., 2018). In addition, the renewed knowledge and optimized management regarding VOD risk factors as well as advances in prophylaxis and treatment have promoted the application of GO (Cortes et al., 2020).

ADC linked with pyrrolobenzodiazepine (PBD) dimer is a new strategy for treatment of various cancers (Hartley, 2021). SGN-CD33A is a pyrrolobenzodiazepine (PBD) dimer-based antibody-drug conjugate targeted to CD33. Pre-clinical studies showed that SGN-CD33A was superior to GO in multi-drug resistant AML cell lines as well as in AML patients with poor risk cytogenetics (Kung Sutherland et al., 2013). In an initial phase I study (NCT01902329) designed to assess the safety of the drug, SGN-CD33A used alone resulted in a CR and CRi rate of less than 30% with minimal dose limiting toxicity (DLT) (Stein et al., 2018). SGN-CD33A in combination with a hypomethylating agent yielded a composite CR and CRi rate of 70% (Fathi et al., 2018). In addition, SGN-CD33A in combination with standard 7 + 3 chemotherapy were being assessed in a phase Ib trial (NCT02326584), achieving an encouraging composite CR and CRi rate of 78% (Erba et al., 2016). In this trial, myelosuppression-related side effects were reported in all patients. None of the patients developed VOD, and the 60-day mortality rate was less than 10%. However, due to high mortality rate in the SGN-CD33A-containing arm, the phase III CASCADE trial was discontinued (By The ASCO Post Staff, 2017).

Lintuzumab is a humanized anti-CD33 antibody with a modest single-agent activity against AML. To improve the efficacy, lintuzumab was conjugated to radioactive elements such actinium-225 and bismuth-213. The radio immune conjugates are undergoing clinical investigation in older patients newly diagnosed with AML for whom standard induction treatment is deemed unfit. Actinium-225-lintuzumab was feasible with myelosuppression and no cases of VOD (Finn LE et al., 2017; Rosenblat et al., 2022). Sequential cytarabine and bismuth-213-lintuzumab produced remissions in patients with AML (Rosenblat et al., 2010). Therefore, the conjugate of lintuzumab and actinium-225 or bismuth-213 represents a reasonable treatment option for patients ineligible for intensive chemotherapy.

## Anti-CD123

CD123 is a cytokine receptor widely overexpressed in multiple hematologic cancer cells, especially in leukemic stem cells (LSCs). AML cells with FLT3-ITD or NPM1 mutations are correlated with significantly increased levels of CD123 as compared to AML cells with wild-type FLT3 or NPM1 (Ehninger et al., 2014; Cortes et al., 2018; Cruz et al., 2018). Overexpression of CD123 is associated with high-risk disease characteristics in adult and pediatric AML. CD123 as an important biomarker can be potentially targeted in relapsed or refractory AML (Lamble et al., 2022).

CSL360 is a chimeric monoclonal antibody directed against CD123 and is believed to inhibit proliferation of AML cells (Roberts et al., 2008). However, CSL360 was far less efficacious in patients with relapsed/refractory or high risk AML (He et al., 2015). CSL362 (talacotuzumab), another monoclonal antibody directed against CD123, demonstrated promising activity in AML patients (Xie et al., 2017). However, talacotuzumab alone or in combination with other drugs showed limited efficacy but considerable toxicity in AML patients in recent clinical study (Kubasch et al., 2020; Montesinos et al., 2021). Many preclinical trials have studied various CD123 antibodies, such as CD123 antagonistic peptide (Xu et al., 2021b; Xu et al., 2022) and protein-CD123 fusion antibodies (Tahk et al., 2021), which significantly reduced BM infiltration or enhanced the elimination of AML-initiating cells. SL-401 is a cytotoxin that fuses the diphtheria toxin (DT) to the IL3 ligand. It was the first treatment approved by the FDA for the management of blastic plasmacytoid dendritic cell neoplasms (BPDCN) in adult and pediatric patients 2 years and older (Jen et al., 2020), with good tolerability and a predictable and favorable safety profile (Pemmaraju et al., 2022). Clinical trials have shown that treatment with SL-401 was associated with reduced drug resistance and better prognosis in AML patients at high risk for relapse. It is worth noting that a serious adverse reaction induced by SL-401 had caused capillary leak syndrome leading to one death, which was reported among the cohort in remission with MRD (American Society of Hematology, 2016; Lane et al., 2016).

## Anti-CD3/CD33 or CD3/CD123 bispecific antibody

Bispecific T-cell-engaging (BiTE) antibodies are composed of dual variable regions that simultaneously bind to CD3 on cytotoxic T lymphocytes and tumor cell antigens on malignant cells, activating the effector function, accompanied by release of cytokines and destruction of tumor cells. By means of this strategy, virtually all T lymphocytes can be recruited and directed against leukemic blasts irrespective of its specificity. The preclinical trial proved that CD3/CD33-directed antibody constructs might be utilized as an alternative treatment in AML (Reusch et al., 2016). AMG 330 has been shown to exhibit strong anti-tumor responses both *in vitro* and in mouse models (Laszlo et al., 2015; Krupka et al., 2016; Jitschin et al., 2018). A clinical trial (NCT02520427) designed to investigate the safety and tolerability of AMG 330 in patients with relapsed/refractory AML is in progress. CD3^−^CD123-BiTE antibody is another promising therapy for patients with relapsed/refractory AML (Bonnevaux et al., 2021). Additional early clinical studies are ongoing to investigate CD3^−^CD123-BiTE antibodies, such as XmAb14045 (NCT02730312), and flotetuzumab (MGD006; NCT02152956), a dual-affinity retargeting (DART) molecule.

## Dendritic cell vaccines therapy

Cancer vaccines represent a groundbreaking therapeutic approach that employs immune effector cells to eradicate malignant cells. Upon activation of immune signaling, presentation of tumor associated antigens is increased, breaking the tolerance to tumor, and at the same time, immune regulation against autoimmunity is maintained. Dendritic cell (DC)-based vaccines offer the potential of a non-toxic and effective immunotherapeutic strategy. The four most common tumor types can be targeted include melanoma, prostate cancer, glioblastoma and renal cell carcinoma (Germeau et al., 2005; Melero et al., 2014; Anguille et al., 2015; Polyzoidis et al., 2015; Schreibelt et al., 2016). The primary types of cancer vaccines undergoing clinical trials for AML patients are DC-based vaccines (Figure 1C).

## WT1 DC vaccine

AML cells have been shown to express the Wilms’ tumor 1 (WT1) protein and upregulation of this protein is a predictor of poor prognosis in AML and MDS patients (Bergmann et al., 1997; Brayer et al., 2015). The specific antibody targeting WT1 shows a good inhibitory effect on AML, which prompted the conduct of a clinical trial for relapsed/refractory AML (NCT0458012) (Augsberger et al., 2021). In addition, TCR-T cell therapy or CRISPR-Cas9 genome editing tools has a potential strategy for WT1-exprssing AML (Tawara et al., 2017; Chapuis et al., 2019; Ruggiero et al., 2022). WT1 is one principal protein studied for vaccination potential and is currently in the clinical investigation phase. In a phase I/II trial, Tendeloo et al. assessed the DC-WT1 vaccine in 10 AML patients who achieved CR or PR following chemotherapy and were at a high risk of relapse (Van Tendeloo et al., 2010). Subsequently, the clinical results of 66 cancer patients after DC-WT1 vaccination, including thirty AML patients who were at very high risk of relapse, were reported (Table 2, NCT00965224), which proved that WT1-targeted DC-based vaccination is an effective immunotherapeutic strategy to prevent relapse in AML patients (Anguille et al., 2017). In another phase I/II trial (NCT01051063), 5 elderly AML patients observed clinical benefit after receiving WT1 recombinant protein vaccination (Kreutmair, 2022). Moreover, clinical trial found the decrease of the regulatory T cells in 8 AML patients following WT1 peptide-loaded DC vaccination (Ogasawara et al., 2022).

**TABLE 2:** Current clinical trials using dendritic cell vaccination for immunotherapy of AML.

Trial identifier	Study name	Conditions	Antigen	Phases	Enrollment	Start date	Completion date	Status
NCT00965224	Efficacy of Dendritic Cell Therapy for Myeloid Leukemia and Myeloma	AML/CML/MM	WT1	Ⅱ	50	2010	2014	Unknown
NCT01686334	Efficacy Study of Dendritic Cell Vaccination in Patients With Acute Myeloid Leukemia in Remission	AML	WT1	Ⅱ	130	2012	2024	Recruiting
NCT04747002	Investigator-initiated Clinical Trial (Phase II) of Cancer Vaccine “Dainippon Sumitomo Phama (DSP)-7888″for Acute Myeloid Leukemia Patients	AML in Remission	WT1	Ⅱ	100	2020	2024	Recruiting
NCT04284228	Antigen-specific T-cell Therapy for AML or MDS Patients With Relapsed Disease After Allo-HCT	AML	WT1/PRAME/Cyclin A1	I/II	22	2020	2023	Recruiting
		MDS						
NCT05000801	Clinical Study of DC-AML Cells in the Treatment of Acute Myeloid Leukemia	AML	WT1/hTERT/Survivin	Not Applicable	20	2021	2026	Recruiting
NCT01096602	Blockade of PD-1 in Conjunction With the Dendritic Cell/AML Vaccine Following Chemotherapy Induced Remission	AML	Multiple	Ⅱ	63	2010	2024	Active, not recruiting
NCT03059485	DC/AML Fusion Cell Vaccine vs. Observation in Patients Who Achieve a Chemotherapy-induced Remission	AML	Multiple	Ⅱ	75	2017	2025	Recruiting
NCT03697707	Efficacy and Safety of Immunotherapy With Allogeneic Dendritic Cells, DCP-001, in Patients With Acute Myeloid Leukaemia (ADVANCE-II)	AML in Remission	Multiple	II	20	2018	2022	Active, not recruiting
NCT03679650	Dendritic Cell/AML Fusion Cell Vaccine Following Allogeneic Transplantation in AML Patients	AML	Multiple	Ⅰ	45	2018	2026	Recruiting

## Human telomerase reverse transcriptase (hTERT) DC vaccine

Telomerase is a ribonucleoprotein enzyme that positively expressed in most malignant tumor cells and is critical for increasing proliferative potential of cancer cells (Counter et al., 1995; Zhang et al., 1996; Ohyashiki et al., 1997; Stewart and Weinberg, 2000). Telomerase can be used as an important predictor for AML due to its strong expression in AML patients, particularly in high-risk cytogenetic AML subtypes. A phase II clinical trial (NCT00510133) enrolled a total of 22 patients achieving early or subsequent complete remission at intermediate risk or high risk of AML (Khoury et al., 2017). The relapse free survival achieved in this trial is superior to that reported in previous trials, but further clinical validation of these results is needed. Preclinical models confirmed the improved survival outcome and showed that telomerase was upregulated in AML blasts, and reduced telomerase activity thwarts leukemic cell proliferation and disease relapse after chemotherapy (Bruedigam et al., 2014). The TERT gene variability and telomere length affect risk and overall survival in AML (Dratwa et al., 2021). It was reported that diverse short telomere-related somatic mutations promote transformation to MDS/AML (Schratz et al., 2021). If the same results can be obtained from further clinical trials, hTERT-DCs should soon become a form of safe and effective immunotherapy that can be used to extend relapse-free survival for AML patients in complete remission. The approach would provide promising treatment options that bring new hope to AML patients.

## DC-AML fusion vaccine

An individualized cancer vaccine strategy was developed by Jacalyn Rosenblatt et al. in which fusion hybrids of autologous dendritic cells and patient-derived AML cells are used to generate antileukemic responses (Rosenblatt et al., 2016). In this phase I/II clinical trial (NCT01096602), a cohort of 17 subjects were responding well to front-line treatment and subsequently received vaccination to target MRD and prevent relapse. The vaccine regimens induced strong T-cell expansion in both PB and BM. Meanwhile, 12 subjects in the cohort undergoing vaccination achieved prolonged survival when followed up to 5 years. These results show that individualized vaccine regimens for AML patients in remission can enhance leukemia-specific T-cell responses and prevent disease relapse.

Cancer vaccines are used to confer protective immunity and enhance tumor antigen-specific T-cell and antibodies. However, clinical outcomes have shown little promise to date. This is largely due to immune suppression in the process of tumor growth, which affects the vaccines’ capability to induce strong and systemic immune responses and functionality of immune responses at the tumor site. Therefore, new approaches have been developed, supported by findings demonstrating that human dendritic cells (DCs) can be cultured *in vitro* (Steinman and Banchereau, 2007). The success of DC vaccines generated enormous enthusiasm in DC vaccines employing the latest technology. Major efforts have often directed to the origin of DCs. For example, specific subsets of naturally occurring DCs are superior to monocyte-derived DCs in terms of potency and cost-effectiveness. Results from preliminary trials demonstrated safety and feasibility of next-generation DC vaccines employing naturally occurring DC subsets; furthermore, this approach has resulted in superior efficacy. A phase I trial showed that 12 elderly patients with late-stage AML receiving a DC vaccine generate both cellular and humoral immune response (van de Loosdrecht et al., 2018). A phase I/II trial (NCT01146262) showed DC-vaccines with leukemic apoptotic bodies produced a safety and efficacy for the AML patients (Chevallier et al., 2021). On the other hand, a major impediment with regard to DC vaccination is to pinpoint the right antigen to be vaccinated for maximal T-cell effector function, stimulation of memory T-cells, and reducing the expansion of inhibitory subsets. Normal cancer antigens such as WT1 and hTERT loading to AML patients’ DCs are currently under clinical investigation. Another method to improve the immunogenicity of DC is to combine with hypomethylating agents (Nahas et al., 2019) or immune checkpoint inhibitors (Stroopinsky et al., 2021). Future development of cancer vaccines would rely more on multiple antigens or individualized neoantigens to maximize vaccine-based immune responses.

## CAR-T cell therapy

CARs, unlike TCRs which interact with HLA molecules, do not need antigen processing or presentation by the HLA peptidome (June et al., 2018), and therefore broadly recognize target antigens independent of HLA background (Figure 1D).

CD19-specific CAR-T cells represent a well-established CAR-T cell therapy with restrictive expression pattern and favorable safety outcome. The promise of anti-CD19 CAR-T cells was immense in ALL, achieving CR in over 90% of B-ALL patients (Davila et al., 2014; Maude et al., 2014). Considering the intense prior treatment: a median of 3 prior intensive chemo regimens with over 1/3 relapsed following allogeneic HSCT, these clinical results are impressive. Anti-CD19 CD28-based CAR-T therapy induced a CR rate of 57% in seven patients with DLBCL with no response to three prior lines of therapy (Locke et al., 2017). The modified CD19 CAR-T cells using the FasT system displayed favorable responses for B-cell ALL in both preclinical study and a phase I trial (NCT03825718) (Yang et al., 2022). CAR-T cell therapy targeting CD19 induces high response rates in B-ALL with central nervous system leukemia (NCT02782351) (Qi et al., 2022). In the context of AML, however, the putative antigens targeted by CAR-T cells must have a restrictive expression pattern to avert destruction of normal myeloid cells. Some targets have been identified in preclinical trials, including natural killer group 2 member D (NKG2D), CD123, CD33, Lewis Y (LeY), CLL-1, etc.

In a phase I study, four patients infused with anti-LeY CAR-T cells were examined to determine the safety and clinical efficacy of adoptive transfer of T cells. The CAR T cells sustained efficacy for up to 10 months post infusion, confirming the feasibility and safety of anti-LeY CAR-T cells in patients with high-risk AML, and demonstrating long-term efficacy (Ritchie et al., 2013). CLL-1 is strongly expressed on AML cells and not detectable on normal HSCs, making it an ideal target in immunotherapy for AML. In a phase I trial that included 10 adult participants with relapsed or refractory AML, chemotherapy coupled with CLL-1 CAR-T cell therapy achieved a CR rate of 70% when followed up to a median period of 173 days, proving that CLL-1 CAR-T cell therapy can mitigate the risk of relapse and improve patient survival (Jin et al., 2022). CLL-1 CAR-T cells with PD-1 knockdown show a successful therapy in patients and represent a feasible immunotherapeutic alternative for treating relapsed or refractory AML (Lin et al., 2021; Ma et al., 2022). Recently, CAR-T cell treatments targeting new potential protein markers (e.g., Siglec-6 and CD70) have been reported. Siglec-6 and CD70 are found on the surface of most AML cells, but are rare or absent in normal BM. Experiments on Siglec-6 and CD70 CAR-T cells in both human and animal models show anti-AML activity and ability to expand and persist (Jetani et al., 2021; Sauer et al., 2021). Based on the CD70 target immunotherapy, the modified CD70-targeted CAR by CD8 non-cleavable hinge method enhance binding capability and expansion, resulting in higher antitumor potency (Leick et al., 2022). Despite the growing number of early-phase studies showing potent antitumor responses of multiple CAR-T cells in recent years, translation into clinical practice and benefits has been slow and challenging. In several early phase clinical trials (Table 3), some adults have received AML CAR-T cell immunotherapy. All NKG2D, CD123, and CD33 have been studied extensively, which are described below.

**TABLE 3:** Current clinical trials using CAR T-cell for immunotherapy of AML.

Trial identifier	Study name	Conditions			Antigen		Phases		Enrollment		Start date		Completion date		Status	
NCT04033302	Multi-CAR T-cell Therapy Targeting CD7-positive Malignancies	AML			CD7		I/II		30		2019		2023		Recruiting	
NCT04762485	Humanized CD7 CAR T-cell Therapy for r/r CD7^+^ Acute Leukemia	AML			CD7		I/II		20		2021		2024		Recruiting	
NCT05377827	Dose-Escalation and Dose-Expansion Study to Evaluate the Safety and Tolerability of Anti-CD7 AllogeneicCAR T-Cells (WU-CART-007) in Patients With CD7^+^ Hematologic Malignancies	AMLetc.			CD7		I		48		2022		2025		Not yet recruiting	
NCT03896854	CART-19 T-cell in CD19 Positive Relapsed or Refractory Acute Myeloid Leukemia (AML)	AML			CD19		I/II		15		2017		2024		Recruiting	
NCT04796441	Clinical Study of Universal CAR-γδT Cell Injection in the Treatmentof Patients With Relapsed AML After Transplantation	AML			CD19		Not Applicable		20		2020		2022		Recruiting	
NCT04257175	CAR-T CD19 for Acute Myelogenous Leukemia With t 8:21 and CD19 Expression	AML			CD19		II/III		10		2020		2023		Recruiting	
NCT05513612	Novel CAR-T Cell Therapy in the Treatment of Hematopoietic and Lymphoid Malignancies	AMLetc.			CD19		I		20		2020		2026		Recruiting	
NCT05388305	Universal CAR-γδT Cell Injection in the AML Patients	AML			CD19		Not Applicable		30		2022		2023		Recruiting	
NCT01864902	Treatment of Relapsed and/or Chemotherapy Refractory CD33 Positive Acute Myeloid Leukemia by CART-33	Relapsed Refractory AML			CD33		Ⅰ/Ⅱ		10		2013		2017		Unknown	
NCT02799680	Allogeneic CART-33 for Relapsed/Refractory CD33^+^ AML	Relapsed Refractory AML			CD33		Ⅰ		12		2015		2018		Unknown	
NCT03971799	Study of Anti-CD33 Chimeric Antigen Receptor-Expressing T Cells (CD33CART) in Children and YoungAdults With Relapsed/Refractory Acute Myeloid Leukemia	AML			CD33		I/II		37		2020		2039		Recruiting	
NCT05008575	Anti-CD33 CAR NK Cells in the Treatment of Relapsed/Refractory Acute Myeloid Leukemia	AML			CD33		I		27		2021		2023		Recruiting	
NCT05105152	PLAT-08: A Study Of SC-DARIC33 CAR T Cells In Pediatric And YoungAdults With Relapsed Or Refractory CD33^+^ AML	AML			CD33		I		18		2021		2039		Recruiting	
NCT04835519	Phase I/II Study of Enhanced CD33 CAR T Cells in Subjects With Relapsed or Refractory Acute Myeloid Leukemia	AML			CD33		I/II		25		2022		2024		Recruiting	
		Relapse Leukemia														
		Refractory AML														
NCT05445765	Anti-CD33 CAR-T Cells for the Treatment of Relapsed/Refractory CD33^+^ Acute Myeloid Leukemia	Relapsed/Refractory AML			CD33		I		10		2022		2024		Not yet recruiting	
NCT05473221	Evaluate the Safety and Efficacy of CD33 CAR-T in Patients With R/R AML	AML			CD33		I		20		2022		2025		Not yet recruiting	
NCT04351022	CD38-targeted Chimeric Antigen Receptor T-cell (CART) in Relapesd or Refractory Acute Myeloid Leukemia	AML			CD38		I/II		20		2017		2023		Recruiting	
NCT05239689	Clinical Study of CD38 CAR-T Cells in the Treatment of Hematological Malignancies	AML			CD38		I		36		2022		2024		Recruiting	
NCT05442580	CART-38 in Adult AML and MM Patients	AML			CD38		I		36		2022		2040		Not yet recruiting	
		MM														
NCT04662294	CD 70 CAR T for Patients With CD70 Positive Malignant Hematologic Diseases	AML			CD70		I		108		2021		2027		Recruiting	
NCT04692948	TAA6 Cell Injection In The Treatment of Patients With Relapsed/Refractory Acute Myeloid Leukemia	AML			CD276		Not Applicable		5		2019		2023		Recruiting	
NCT02159495	Genetically Modified T-cell Immunotherapy in Treating Patients WithRelapsed/Recurrent Blastic Plasmacytoid Dendritic Cell Neoplasm	AML			CD123		Ⅰ		31		2015		2022		Active, not recruiting	
NCT03114670	Donor-derived Anti-CD123-CART Cells for Recurred AML After Allo-HSCT	Adult AML			CD123		Ⅰ		20		2017		2021		Unknown	
NCT03190278	Study Evaluating Safety and Efficacy of UCART123 in Patients With Acute Myeloid Leukemia	AML			CD123		Ⅰ		65		2017		2023		Recruiting	
NCT03556982	CART-123 FOR Relapsed/Refractory Acute Myelocytic Leukemia	Relapsed Refractory AML			CD123		Ⅱ/Ⅲ		20		2018		2020		Unknown	
NCT04265963	CD123-Targeted CAR-T Cell Therapy for Relapsed/Refractory Acute Myeloid Leukemia	AML			CD123		I/II		45		2019		2022		Recruiting	
NCT04272125	Safety and Efficacy of CD123-Targeted CAR-T Therapy for Relapsed/Refractory Acute Myeloid Leukemia	AML			CD123		I/II		40		2019		2023		Recruiting	
NCT04318678	CD123-Directed Autologous T-Cell Therapy for Acute Myelogenous Leukemia (CATCHAML)	AMLetc.			CD123		I		32		2020		2025		Recruiting	
NCT04678336	CD123 Redirected T Cells for AML in Pediatric Subjects	AML in relapse			CD123		I		12		2021		2036		Recruiting	
		AML pediatric														
		AML refractory														
NCT04884984	Anti-CLL1 CAR T-cell Therapy in CLL1 Positive Relapsed/Refractory Acute Myeloid Leukemia (AML)	AML			CLL1		I/II		20		2017		2024		Recruiting	
NCT05467202	Evaluate the Safety and Efficacy of CLL1 CAR-T in Patients With R/R AML	AML			CLL1		I		20		2022		2025		Not yet recruiting	
NCT04219163	Chimeric Antigen Receptor T-cells for The Treatment of AML Expressing CLL-1 Antigen	AML			CLL1		I/II		18		2020		2038		Recruiting	
NCT04923919	Clinical Study of Chimeric Antigen Receptor T Lymphocytes (CAR-T) in the Treatment of Myeloid Leukemia	AML			CLL1		I		100		2021		2023		Recruiting	
NCT05252572	Clinical Study of CLL1 CAR-T Cells in the Treatment of Hematological Malignancies	AML			CLL1		I		36		2022		2024		Recruiting	
NCT05023707	Anti-FLT3 CAR T-cell Therapy in FLT3 Positive Relapsed/Refractory Acute Myeloid Leukemia	AML			FLT3		I/II		5		2021		2025		Recruiting	
NCT05432401	TAA05 Injection in the Treatment of Adult Patients With FLT3-positive Relapsed/Refractory Acute Myeloid Leukemia	FLT3-positive Relapsed/Refractory AML			FLT3		I		18		2022		2025		Recruiting	
NCT05445011	Anti-FLT3 CAR-T Cell (TAA05 Cell Injection) in the Treatment of Relapsed/Refractory Acute Myeloid Leukemia	AML			FLT3		I		12		2022		2027		Recruiting	
NCT03018405	A Dose Escalation Phase I Study to Assess the Safety and Clinical Activity of Multiple Cancer Indications	MDS/AML/MM			NKG2D		Ⅰ		146		2016		2021		Unknown	
NCT04658004	NKG2D CAR-T Cell Therapy for Patients With Relapsed and/or Refractory Acute Myeloid Leukemia	AML			NKG2D		I		36		2021		2027		Not yet recruiting	
NCT04599543	IL3 CAR-T Cell Therapy for Patients With CD123 Positive Relapsed and/or Refractory Acute Myeloid Leukemia	AML			IL3		I		36		2020		2026		Not yet recruiting	
NCT05266950	Safety and Efficacy Study of CI-135 CAR-T Cells in Subjects With Relapsed or Refractory Acute Myeloid Leukemia	AML			CI-135		I		7		2021		2023		Recruiting	
NCT04803929	Clinical Study of Anti-ILT3 CAR-T Therapy for R/R AML (M4/M5)	AML			ILT3		I		25		2021		2026		Recruiting	
NCT05463640	Evaluate the Safety and Efficacy of ADGRE2 CAR-T in Patients With R/R AML	AML			ADGRE2		I		20		2022		2022		Not yet recruiting	
NCT05488132	Administration of Anti-siglec-6 CAR-T Cell Therapy in Relapsed and Refractory Acute Myeloid Leukemia (rr/AML)	Refractory/Relapse AML			Siglec-6		I/II		20		2022		2025		Recruiting	
NCT03795779	CLL1-CD33 cCAR in Patients With Relapsed and/or Refractory, High Risk Hematologic Malignancies	HM			CLL1/CD33		I		20		2018		2022		Recruiting	
		AML														
		MSD														
		CML														
NCT05016063	Dual CD33-CLL1-CAR-T Cells in the Treatment of Relapsed/Refractory Acute Myeloid Leukemia	AML			CD33/CLL1		I		32		2021		2023		Not yet recruiting	
NCT05248685	Optimized Dual CD33/CLL1 CAR T Cells in Subjects With Refractory or Relapsed Acute Myeloid Leukemia	AML			CD33/CLL1		I		20		2022		2024		Recruiting	
NCT05467254	Evaluate the Safety and Efficacy of CLL1+CD33 CAR-T in Patients With R/R AML	AML			CLL1/CD33		I		20		2022		2025		Not yet recruiting	
NCT04010877	Multiple CAR-T Cell Therapy Targeting AML	AML			CLL1, CD33/CD123		I/II		10		2019		2023		Recruiting	
NCT03222674	Multi-CAR T-cell Therapy for Acute Myeloid Leukemia	AML			Multiple		Ⅰ/Ⅱ		10		2017		2020		Unknown	
NCT03291444	CAR-T Cells Combined With Peptide Specific Dendritic Cell in Relapsed/Refractory Leukemia/MDS	AML			Multiple		I		30		2017		2025		Recruiting	
NCT04766840	Donor-derived CAR-T Cells in the Treatment of AML Patients	AML			Multiple		I		9		2021		2023		Not yet recruiting	

## NKG2D CAR-T

NKG2D ligands are selectively expressed on monoblastic cells in AML, but absent or weakly expressed on myeloblastic cells and chemotherapy-resistant leukemic stem cells in AML, which avoid NK-mediated killing and cause immune evasion (Diermayr et al., 2008; Paczulla et al., 2019). Therefore, inhibitor-mediated activation of surface NKG2D ligands is a useful approach to immunotherapy of AML. Based on this, bispecific FLT3 antibody system (scFv)/NKG2D-CAR T cells have been designed, effectively eradicating AML cells in preclinical study (Li et al., 2022). NKG2D CAR and IL-15 constitutively expressed enhance the efficacy and significantly prolong the mouse survival in the KG-1 AML model.

The safety and lymphodepleting conditioning chemotherapy in participants with AML/MDS or relapsed/refractory multiple myeloma were assessed in a phase I trial (NCT02203825) with dose escalation design (Baumeister et al., 2019). A NKG2D CAR was constructed by human NKG2D linked to CD3ζ signaling domain. Following the treatment, no dose-limiting toxicities, cytokine release syndrome, or neurological toxicities associated with CAR T cells were reported. No autoimmune disorders or serious adverse reactions were found to be associated with NKG2D-based CAR-T cells. NKG2D CAR-T cells were not expanded or persisted in the majority of patients, which is in agreement with murine models in which repeat infusions are needed to eliminate the tumor completely (Barber et al., 2011). As a highly conserved receptor, NKG2D is less likely to elicit immune responses. Additional CAR trials (NCT03018405) assessing multiple infusions and higher doses are underway to further establish the safety profile of NKG2D CAR therapy (Sallman et al., 2018).

## CD123 CAR-T

CD123 is a poor prognostic antigen marker found on AML cells and chemo-resistant leukemic stem cells. Anti-CD123 CAR-T therapy was able to eradicate AML blasts in experiments (Arcangeli et al., 2017). Gene-edited CAR-T cells targeting CD123 are primarily directed against AML cells, with tolerable toxicity to normal cells (Sugita et al., 2022).

An early phase I pilot study sponsored by the University of Pennsylvania (NCT02623582) sought to evaluate autologous T lymphocytes containing anti-CD123 linked to TCR/4-1BB domains in AML patients (Tasian, 2018). Five adult subjects with relapsed/refractory AML were given lymphodepleting chemotherapy prior to the CD123 CAR-T cells infusion. However, this trial was eventually terminated because the regimen had induced little anti-leukemic efficacy and on target/off tumor toxicologic effects had been reported. Another phase I trial also reported preliminary results using lentiviral infected T cells to express a CAR targeting CD123 (NCT02159495). CD123-based CAR-T cell treatment induced complete remission in some participants, who became eligible for a second HSCT. Moreover, other drugs such as demethylating agents can augment immune responses and facilitate clearance of tumor cells by CD123-targeting CAR-T cells (El Khawanky et al., 2021). New CD123-directed CAR-T cells were engineered by the rapidly switchable universal CAR-T platform and retain complete anti-AML potency as well as ensuring improved safety of CD123-based immunotherapy in different applications. Currently, a dose-escalating clinical trial is ongoing to assess the therapeutic benefit of this new study drug (NCT04230265) (Loff et al., 2020). Other clinical trials for CD123 CAR-T cells are ongoing including NCT03766126, NCT03114670, NCT03190278, and NCT03631576.

## CD33 CAR-T

The CD33 antigen exhibits high expression levels in AML blasts; however, normal myeloid progenitors also express CD33 antigen, limiting its potential as an immunotherapeutic target for AML (Ehninger et al., 2014). An *in vivo* NSG mice model experimenting with AML xenotransplantation has reported significant reduction of leukemic burden and prolonged survival (O'Hear et al., 2015).

Worldwide, several other trials of anti-CD33 CAR T cells for the treatment of AML are underway. A safety test of varying doses of CD33-CAR-T cells in patients with CD33^+^ AML was conducted by the MD Anderson Cancer Center (MDACC) (NCT03126864). The results found that CAR-T cell production was only possible in cohorts with increased lymphocyte levels and decreased blood cells, but along with systemic inflammatory syndrome and neurotoxic effects (Tambaro et al., 2021). Chinese PLA General Hospital is conducting an early phase I trial (NCT01864902) and will assess the safety and feasibility of CD33-engineered lymphocyte therapy in patients with AML that is relapsed or not responding to chemotherapy. After initial treatment, one participant with relapsed AML who had previously received an infusion of CD33 CAR T cells had a noticeable decrease in blast count in BM; however these AML blasts subsequently recovered, and relapse occurred 2 months post infusion (Wang et al., 2015). A preclinical experiment evaluated six CD33 CAR constructs using scFv containing CD3ζ domain and showed antileukemia activity, which results in a clinical trial for relapsed/refractory childhood AML (NCT03971799) (Qin et al., 2021). Additional clinical trials with CD33-CAR-T cell strategies are underway (NCT03927261).

To achieve optimal therapeutic effect, the preferred marker in CAR-T treatment should be present in the majority of cancer cells including the subpopulation of cancer stem cells in a substantial percentage of patients. Moreover, to avoid undesired toxicity, the candidate target should not be present on normal tissues or on CAR T cells to avert unwanted self-elimination of CAR T cells. Without a favorable target such as CD19, it is of critical importance to develop a generalizable and combinatorial targeting approach that allows identification of several promising target pairings in AML CAR-T therapy. It was found that AML cells were associated with overexpression of CD33/TIM3 and CLL1/TIM3 as compared to normal tissues when analyzing the density of CD33, CD123, TIM3, among other antigens, on AML bulk and LSCs at initial diagnosis and relapse (Haubner et al., 2019). Like bispecific antibody, CD33/TIM3 or CLL1/TIM3 may serve as viable targets to improve treatment efficiency while limiting toxicity during CAR-T therapy in AML patients. Meanwhile, CD33/CLL-1 is a preferential generic combinatorial immunotarget in pediatric AML (Willier et al., 2021). Moreover, the combination of CAR-T cells and other drugs such as targeted/chemotherapy agents has synergistic effect on AML (El Khawanky et al., 2021; Li et al., 2022). Also, the feasibility of constructing an antigen specific to AML can be considered by removing CD33 from normal cells to overcome resistance to CD33-directed therapy and realize on-target effects with CAR-T cells (Kim et al., 2018).

## TCR-T cell therapy

Similar to CAR-T cells, TCR-T cells is belonged to adoptive cell therapy with antigen-specific T cells, and was successfully adopted and transferred in mice in 1986. TCR-T cell therapy targeting New York esophageal squamous cell carcinoma (NY-ESO)-1 has achieved remarkable clinical benefits in the treatment of solid tumors (D'Angelo et al., 2018; Robbins et al., 2011). TCR-T cell specific for MAGE-A4, HA-1H, WT1 are potential strategies for hematological malignancies (Kageyama et al., 2015; Tawara et al., 2017; van Balen et al., 2020; Vasileiou et al., 2021).

In several clinical trials, WT1-specific TCR-T cells has shown strong clinical responses with tolerated safety for AML patients (Tawara, I. et al. S, 2017). Moreover, numerous clinical trials are underway to evaluate other antigen-specific TCR-T cells in AML. Recently, Kang et al. reviewed the update and challenges of TCR-T for AML. TCR-T exhibits a promising tool for AML patients according to the benefit efficacy of clinical trials. However, the toxicity produced in the treatment process and the persistence of *in vivo* TCR-T cells should be the urgent problems of TCR-T immunotherapy.

## Conclusion

In this review, we summarized the recent advances for AML immunotherapies and noted the representative emerging strategies. The immunotherapy as a treatment option for AML patients with relapse has shown great promise. However, several limitations to the AML immunotherapy are limited its application in AML. Immune evasion remains a clinical challenge in effectively implementing immunotherapeutic strategies. AML is a complex disorder associated with distinctive genetic features and accumulation of genetic aberrations. The lower level of mutation load in AML would lead to higher chance of immune escape of the immune system and less neoantigen epitopes when compared to melanoma and other types of solid cancers such as lung cancer. Considering reduced mutation load, a core issue around AML requiring further exploration is the extent of immunogenicity. Therefore, clinical success of immune therapy in AML will depend on at least the following efforts: exploiting immune escape mechanism and identifying specific targets.

AML has a distinct pattern of development and progression, and there is a paucity of data on the mechanisms of immune activation or tolerance. The mechanisms underlying antitumor immune responses are well understood and the strategies of immune evasion employed by solid tumors have been studied extensively. Accumulating evidence suggests that certain immune evasion mechanisms are exclusive to AML despite the fact that PD1/L1, among other immune escape pathways, are shared with solid malignancies. Although currently encountering various obstacles, the progress in exploring the mechanisms by which immune surveillance and evasion take place in AML can be attained *via* genetically modified AML models reconstructing the disease. Moreover, integrated data on antigens coupled with analytical platforms to screen out truly AML-specific antigen markers are required. Up till now, transcriptome analyses are mainly used for screening candidate AML targets assuming a direct link between mRNA abundance and protein expression. This complex correlation cannot represent the complete set of proteins expressed by the cell, due to numerous factors such as variations in drug metabolism and mechanisms of post-transcriptional regulation. Therefore, integrative analysis of RNA transcripts and proteins should be performed to complement mRNA or protein expression analysis. In addition, coupling advanced proteomics analytical approaches with plasma membrane proteome enrichment will facilitate direct assessment of surface proteins. In summary, because of the heterogeneous nature of AML, each patient may present distinct cytogenetic aberrations and chromosomal rearrangements that require biomarker-specific individualized treatment. Therefore, tailored treatments combining different forms of immunotherapy with chemotherapy and autologous or allogeneic SCT hold immense promise.
